# Carcass and meat characteristics of male Kacang goat fattened by complete silage

**DOI:** 10.14202/vetworld.2020.706-715

**Published:** 2020-04-17

**Authors:** Paulus Klau Tahuk, Gerson F. Bira

**Affiliations:** Department of Animal Science, Faculty of Agriculture, Universitas Timor, Kefamenanu, North Central Timor, East Nusa Tenggara, Indonesia

**Keywords:** carcass characteristics, chemical and physical quality of meat, complete silage, fattening, male Kacang goat

## Abstract

**Aim:**

The aim of the study was to determine the carcass and meat characteristics of male Kacang goat fattened by complete silage made from natural grass, *Sorghum bicolor* (L.) Moench and *Pennisetum purpureum*.

**Materials and Methods:**

This study examined 12 young male Kacang goats aged 10-12 months with an initial body weight of 10-12 kg. The livestock was divided into three groups randomly to receive feed treatments. The three treatments of this study included T1: Complete silage made from natural grass; T2: Complete silage made from *S. bicolor* (L.) Moench, and T3: Complete silage made from *P. purpureum*. Data were analyzed according to the analysis of variance procedure.

**Results:**

The carcass percentage of livestock T2 and T3 treatment was relatively similar but higher (p<0.05) than the T1 group. The non-carcass percentage of T2 and T3 was relatively the same but lower than T1. The water content of T1 treatment was higher (p<0.05) than T3, but relatively the same as T2 treatment. The collagen content of T2 and T3 was relatively the same but lower (p<0.05) than T1; likewise, the cholesterol of T2 and T3 treatments was relatively similar but higher than T1 treatment. The variables of slaughter and carcass weight, and non-carcass weight, meat protein content, acidity, cooking losses, water holding capacity, and tenderness were not significantly different between groups of animals.

**Conclusion:**

The use of *S. bicolor* (L.) Moench and *P. purpureum* as a basic forage in making complete silage has been shown to increase carcass percentage, the content (%) of fat, collagen, and cholesterol. Otherwise, the treatments have no effect on the content of pH, tenderness (kg/cm^2^), cooking loss (%), and water holding capacity (%) of male Kacang goat fattened. Therefore, *S. bicolor* (L.) Moench and *P. purpureum* plants have great potential to be developed by farmers/ranchers as feed for goats.

## Introduction

Goat livestock productivity is generally determined by many factors, such as feed, management, and animal genetic. The optimal combination of these three factors positively impacts on improving the performance of goats. The feed factor is a component that plays an important role and has a large economic impact on the optimal/absence of raising processes. In addition, the feed cost incurred by farmers to meet the needs of livestock for feed reaches 80% of the total cost of production [1,2].

According to various reports, the cause of the low production of goat meat is the slow pace of livestock development (especially in fiber and dairy goats) and poor management conditions. In addition, nutritional factors also affect the growth of goats, including the types of grassland and their availability, grazing competition, and the number and types of supplements provided [3].

Therefore, providing adequate feed can spur livestock growth, which, in turn, can increase carcass production and meat quality of male Kacang goat fattened. According to Ngadiyono *et al*. [4], a better level of feed consumption in livestock will have a direct effect on increasing growth and optimal body tissue synthesis. As a result, the slaughter weight produced by livestock is higher.

Although the aspect of feed is very important to consider in raising livestock, the reality faced by breeders in the tropics is the high fluctuation of feed between the rainy season and the dry season. In the rainy season, the abundant forage production will have a positive impact on improving livestock performance. Conversely, in the dry season, the availability of feed is very limited, as indicated by lower/negative growth of livestock [5].

In arid climates that occur in Timor Island (West Timor), it is important to develop feed technology, including making complete silage. This is to ensure the sustainability of livestock production, including Kacang goat livestock throughout the year. A complete feed is one of the solutions that can be taken to meet the needs of livestock. The complete feed has complete nutritional content to meet livestock needs. In addition, the application of complete feed technology can also overcome the scarcity of feed in the dry season. According to Ginting [6], viewed from the aspect of metabolism as well as from the perspective of the potential and optimization of the utilization of feed resources, the prospect of using the complete feed in goats is actually quite promising. Wherein metabolically, the energy needs and capacity digestive organs of goat basically require the type of feed with high nutrient concentrations as well as the characteristics of complete feed.

The potential of complete feed to provide solutions to feed fluctuations in the tropics is quite promising. However, the adoption of feed preservation technology by farmers/ranchers is still low. The pattern of livestock raising which is still traditionally extensive is one of the limiting factors. In addition, the goat livestock business that is still part time is one of the factors causing the low development of complete silage adoption technology.

The testing of complete silage technology based on natural grass, *Sorghum bicolor* (L.) Moench and *P. purpureum* on fattening of Kacang goat is one of the solutions adopted to see its impact on livestock performance. In addition, this complete feed trial also aims to find out the potential for its development and the possibility of its application in farmers/ranchers.

This study aimed to determine the effect of giving complete silage based on different forages on the carcasses and meat characteristics of male Kacang goats fattened.

## Materials and Methods

### Ethical approval

The use of male Kacang Goats in this study does not require approval of the institutional ethics committee because there is no invasive treatment for the animals used.

### Study period, location, livestock, feed, and research design

The study was conducted at the Faculty of Agriculture, Universitas Timor, from March to October 2019, which consisted of several stages, namely, preparation, data collection, analysis, and reporting. An analysis of the chemical composition of the feed sample and complete silage ingredients was carried out at the Feed Chemistry Laboratory, Faculty of Animal Husbandry, Universitas Nusa Cendana. Meanwhile, an analysis of the meat physical and chemical quality was carried out at the Meat Science and Technology Laboratory of the Faculty of Animal Sciences, Universitas Gadjah Mada, and analysis of meat cholesterol was carried out at the Nutrition Biochemical Laboratory, Faculty of Animal Sciences, Universitas Gadjah Mada.

This study examined 12male Kacang goats aged 10-12months, with an initial body weight of 10-12kg. The livestock were purchased from the Wini and Ponu regions, North Central Timor.

The feed ingredients used were natural grass, *S. bicolor* (L.) Moench, *P. purpureum*, *Leucaena leucocephala*, corn flour, and bran pollard. All feed ingredients were arranged and processed into rations in the form of complete silage according to the treatment. In addition, the livestock were given mineral premixes to avoid mineral deficiencies that they might experience. The ration was prepared on the basis of the needs of young goats weighing 10kg [7]. The expected daily body weight gain (ADG) was 75g/head/day.

The composition of chemical feed ingredients and complete silage of research (basic of dry matter/DM) is listed in Table-1, while the chemical composition of silage completer is listed in Table-2; and the composition of the ration is shown in Table-3[8].

**Table-1:** The chemical composition of the complete silage constituents^1^.

Nutrient composition	Feed ingredients					

	*Sorghum bicolor (L.) Moench*	*Leucaena leucocephala*	*Pennisetum purpureum*	Natural grass	Corn flour	Bran pollard
Dry matter (%)	29.51	25.60	24.16	26.81	85.633	88.058
Organic matter (%)	88.823	84.081	83.100	76.413	83.159	84.563
Crude protein (%)	10.537	25.059	11.274	4510	9240	17.043
Crude fiber (%)	23.320	18.366	26.342	32.133	3437	7114
Extract ether (%)	2.554	5.514	4.207	1.182	4.640	4.717
CHO (%)	75.732	53.509	67.620	70.721	69.279	62.803
NFE (%)	52.412	35.143	41.278	38.589	65.841	55.690
TDN (%)	84.084*	54.125*	55.484*	55.625*	76.422*	74.235*
GE: MJ/kg DM	16.527	17.212	15.866	13.731	15.808	16.605
Kcal/kg DM	3934.95	4097.99	3777.56	3269.31	3763.74	3953.55
ME (Kcal/kg DM)	2862.60	2945.62	2566.48	1938.44	3553.00	3431.78

1 Results of Feed Chemistry Laboratory Analysis, Faculty of Animal Husbandry Undana (2019); NFE=Nitrogen-free extract, TDN=Total digestible nutrients; GE=Gross energy; ME=Metabolism energy;

* In accordance with the equation Hartadi *et al.* [8]

**Table-2:** The chemical composition of the complete silage^1^.

Treatments	Nutrient composition (%)											

	DM (%)	OM	CP	CF	EE	CHO	NFE	ASH	TDN*	GE		ME
		
	% of DM									MJ/kg DM	Kcal/kg DM	Kcal/kg DM
T1^2^	37.361	86.771	9.895	27.264	5.236	71.595	44.331	13.229	82.00	16.581	3947.920	2731.393
T2^3^	33.793	88.148	13.818	14.867	7.297	67.033	52.167	11.852	78.00	17.435	4151.223	3382.528
T3^4^	32.491	84.841	11.112	22.051	6.981	66.748	44.697	15.159	76.00	16.622	3957.663	2940.450

1 Results of Feed Chemistry Laboratory Analysis, Faculty of Animal Husbandry, Nusa Cendana University (2019);

2 T1=45% Natural grass + 20% *L. leucocephala* + 25% corn flour + 10% bran pollard;

3 T2=45% *S. bicolor (L.) Moench* + 20% *L. leucocephala* + corn flour 25% + 10% bran pollard;

4 T3=45 % *P. purpureum*+ 20% *L. leucocephala* + 25% corn flour + 10% bran pollard; DM=Dry matter; OM=Organic matter; CP=Crude protein; CF=Crudefiber; EE=Extract ether; NFE=Nitrogen-free extract; TDN=Total digestible nutrients; GE=Gross energy; ME=Metabolism energy.

* In accordance with the equation Hartadi*et al.* [8]. *L. leucocephala*=*Leucaena leucocephala*, *S. bicolor*=*Sorghum bicolor*, *P. purpureum=Pennisetum purpureum*

**Table-3:** Composition of research rations (basic DM) [8].

Treatment/feedstuff	The proportion of feed ingredients (%)	Nutrient composition	

		CP ration (%)	TDN ration (%)
T1			
Natural grass	45	5.0085	26.10
leucocephala	20	5.13	15.96
Flour corn	25	1.9725	20.94
Bran pollard	10	1.6412	7.48
Total	100	13.75	70.49
T2			
*Sorghum bicolor* (L.) Moench	45	5.40	26.96
*Leucaena leucocephala*	20	5.13	15.96
Flour corn	25	1.97	20.94
Bran pollard	10	1.6412	7.48
Total	100	14.14	71.33
T3			
*Pennisetum purpureum*	45	5.61	25.65
*Leucaena leucocephala*	20	5.13	15.96
Flour corn	25	1.97	20.94
Bran pollard	10	1.64	7.48
Total	100	14.36	70.03

DM=Dry matter, CP=Crude protein, TDN=Total digestible nutrients

The study used a completely randomized design, with three treatment ration groups and each group consisted of four goats to make total of 12 goats. The composition of the treatment given to the livestock is as follows:

T1:45% Natural grass + 20% *L. leucocephala* + 25% corn flour + 10% bran pollard

T2:45% *S. bicolor* (L.) Moench + 20% *L. leucocephala* + corn flour 25% + 10% bran pollard

T3:45 % *P. purpureum* + 20% *L. leucocephala* + 25% corn flour + 10% bran pollard

The equipment used in this study was 12 individual cages with a size of 70×150cm which was equipped with separate feed and drinking water containers. The other equipment was the digital livestock scales, rougweight brand with a sensitivity of 0.1kg for weighing goats, feed scales capacity 2kg with a sensitivity of 10g, knives, and buckets for concentrate containers. In addition, there were also a Wiley mill diameter filter 1mm for grinding feed samples, a unit of proximate analysis tool, laboratory equipment for analyzing the physical and chemical composition of meat, and meat cholesterol.

### Variables, data collection, and research procedures

#### Research variable

The variables observed in this study included (a) slaughter weight, (b) hot or fresh carcass weight (kg), (c) hot carcass percentage (%), (d) non-carcass weight and percentage, and (e) chemical and physical composition of meat.

#### Data collection and research procedures

The initial stage of this research implementation was the making of complete silage. Natural grass/*S. bicolor* (L.) Moench/*P. purpureum* was harvested early in the flowering period and chopped with a length of ±3-5cm. The chopped *S. bicolor* (L.) Moench/*P. purpureum*/natural grass was then spread on tarpaulin and mixed with *L. leucocephala* which was chopped to the same size.

The next step was additives in the form of corn flour and pollard bran weighed according to the dose and spread evenly over the chopped forage *S. bicolor* (L.) Moench/*P. purpureum*/natural grass and *L. leucocephala*. The mixture of chopped ingredients and additives was stirred until it was evenly distributed. Furthermore, the mixture of feed ingredients was gradually inserted into the fermentor silo (plastic drum), while it was compacted to release as much oxygen as possible.

Silo (plastic drum) that has been filled with the complete silage was stored indoor at room temperature (25°C) for 21days. After 21days, the silo was opened and the observations on the physical and chemical quality of the complete silage were conducted. The observations were aimed at proving that the silage made was in good quality.

The livestock were adapted with complete silage for 14days (2weeks). The goal was to obtain a stable body condition of livestock during the study. In addition, it was also done to eliminate the effects of the previous feed. During the adjustment phase, the livestock were given Wormectin (Production of Medion, Bandung, Indonesia) at a dose of 0.5ml/25kg/day intramuscularly. The aim was to prevent external and internal parasites attacks on the livestock during observations.

### Measurement of carcass characteristics

Slaughter weight of the livestock was obtained from weighing the goat before slaughtering and fasting for 12h; hot or fresh carcass weight (kg) was the result of weighing carcass after being slaughtered, which was obtained from the difference in slaughter weight (kg) and non-carcass weight (kg). The percentage of the hot carcass was a calculation based on the ratio of the weight of the hot carcass (kg) to the slaughter weight (kg) multiplied by 100%.

### Determination of the chemical composition of the meat

The measurement of the chemical composition of meat was done using the near-infrared spectroscopy method by utilizing the FoodScan Meat Analyzer [9].

### Procedure for determination

The samples were ground (milled) using a meat grinder; then, the sample was weighed (±30g); then, the sample inserted into the cup/sample cups (diameter 15cm); and then, the surface was leveled (the surface was sealed); next, the computer connected with the foods scan tool was turned on; the icon for food scan was clicked or “the menu” was selected, and the food scan program was launched; the configuration was selected by specifying parameters of the test including fat, protein, collagen, and water with the wavelengths set up between 800 and 1400nm. The next stage was that the sample cup was inserted into the food scan space; the food scan analysis tool was selected and activated by pressing “Run/On.” After 15min, the food scan analysis had been detected and had read the average moisture, protein, fat, and collagen in % units. In the end, the sample was given a unique code and file that had been read was saved. The measurement of the meat cholesterol levels was done by utilizing the Liebermann–Burchard method [10] and reading using a spectrophotometer at a wavelength of 680nm.

### Determining the physical quality of meat

#### Determining water holding capacity (WHC)

The stage of measurement began with determining free water content (FWC) [11]. Measurement procedures followed as: Weighed ±0.3g sample, placed it on a filter paper. Pressed between two plates under a load of 35kg for ±5min. Established the image area with plastic transparency and determined the specific area (cm^2^). The equation to calculate WHC was as follows: mgH_2_0 = volume of the wet area (cm^2^)/0.0948. FWC (%) was calculated using the following equation: mgH_2_0/sample weight (mg)×100%. To determine the total water content (TWC), the following procedure was used: Weighed filter paper (initial weight), weighed the sample weight ±1g (sample weight), and wrap it with filter paper. Next, preheated the oven, then heated the sample at 110°C for 8h. Weighed it after removing from the oven (final weight). The TWC (%) was calculated using the following equation: Sample weight − (final weight−initial weight)/sample weight (mg) × 100%. The WHC (%) was calculated using the following equation: TWC (%) − FWC (%).

#### Determination of meat tenderness (kg/cm^2^) (Shear force Warner-Bratzler method)

Measurement procedure

Weighed 20g sample, placed it in polypropylene plastic, and packed with a vacuum pack. The sample was heated in a water bath at 80°C for 30min. Next, the sample was removed from the water. After cooling it down, determined a sample field of the size of 1.5×0, 67cm or 1×1cm or form a tube measuring a certain area in the direction of the fibers of the meat. Placed the test sample plate on the tool with the fiber direction in a transverse position and turned on the Warner-Bratzler shear machine. The tool cut the meat fibers. Noted the measurement result. Repeated 2 or 3times [11,12].

#### Determination of the degree of acidity (pH)

Measurement procedure

Samples of meat were mashed by chopping or with a meat grinder. Weighed 2g of the mashed/ground meat for a sample and then diluted the sample with 18ml of distilled water, stirred/mixed until homogeneous, and then filtered it. The following steps were done using a pH meter calibrated using buffer solutions of pH4 and pH7. The filtrate samples were measured by a pH meter with specification HI 9811XPiccolo, Hanna instrument, and the measurements were recorded. Using the samples, measurements were duplicated; after each pH measurement, the pH meter was checked for standardized calibration with solutions of buffer pH7 and pH4 [11].

#### Determining cooking loss

Measurement procedure

Weighed ±20g sample and placed it in plastic polypropylene. Packed samples using a vacuum pack, then heated in a water bath at 80°C for 30min. Determined the cooking loss (%) by the equation A−B/A×100%, where A and B, respectively, were the sample weights before and after heating (g) [13].

### Data analysis

Data were analyzed by one-way analysis of variance. If there were differences among the treatments, further tests were conducted by Duncan’s multiple range test (DMRT) [14]. SPSS software version20 (IBM, NY, USA) was used to facilitate the analysis.

## Results

### Carcass characteristics

Slaughter and carcass weights (kg) of male Kacang goat fattened that obtained complete silage made from natural grass, *S. bicolor* (L.) Moench, and *P. purpureum* were relatively similar between treatments (p>0.05). On the contrary, the carcass percentage of T2 and T3 treatment was not significant and was higher (p<0.05) than the T1 treatment (Table-4).

**Table-4:** Characteristics of carcasses of male Kacang goats fed complete silage.

Variable	T1	T2	T3
Slaughter weight (kg)^ns^	13.25±2.53	15.23±1.96	13.98±1.63
Carcass weight (kg)^ns^	4.26±1.00	5.65±0.82	5.05±0.70
Carcass percentage	32.12±2.00^a^	36.34±1.82^b^	36.07±1.29^b^
Non-carcass weight (kg)^ns^	8.98±1.59	9.88±1.21	8.93±0.95
Non-carcass percentage	67.88±2.00^b^	63.66±1.82^a^	63.93±1.29^a^

Data are presented in average ±SD;

ab different superscript in the same row shows differences (p<0.05),

ns not significant

The non-carcass weight (kg) of male Kacang goats which obtained complete silage made from forages was relatively similar between treatments. However, the non-carcass percentage showed a significant difference between the treatments. Male Kacang goat in T1 treatment had higher non-carcass percentage (p<0.05) than T2 and T3 treatments.

### Meat chemical and physical quality

Meat protein content (%) of male Kacang goat feedlot in T1, T2, and T3 treatment was relatively the same and was still in the normal range (Table-5). The fat content (%) of male Kacang goat in T3 treatment was higher than T1 (p<0.05). In contrast, the livestock in the T2 treatment were relatively the same as T1 and T3 (Table-5).

**Table-5:** Meat chemical and physical quality of male Kacang goat feedlot fed completes silage.

Variable	T1^1^	T2^2^	T3^2^
Meat chemical quality			
Protein content^ns^ (%)	20.41±0.77	20.75±0.72	19.99±0.68
Fat content (%)	3.76±1.36^a^	4.78±1.08^ab^	5.89±1.27^b^
Moisture content (%)	72.72±0.94^b^	71.62±0.49^ba^	71.94±0.86^a^
Collagen content (%)	2.13±0.21^b^	1.78±0.07^a^	1.88±0.12^a^
Cholesterol content (mg/100 g)	35.55±1.16^a^	38.08±1.05^b^	38.53±1.46^b^
Meat physical quality			
pH^ns^	5.45±0.17	5.28±0.43	5.38±0.17
Tenderness (kg/cm^2^)^ns^	8.70±1.25	7.96±0.97	7.19±0.93
Cooking losses (%)^ns^	37.27±1.29	36.51±0.97	35.74±0.93
Water holding capacity (%)^ns^	34.67±1.29	33.91±0.97	33.90±1.17

Data are presented in average ±SD; ^ab^different superscript in the same row shows differences (p<0.05),

ns not significant, T1=45% Natural grass + 20% *L. leucocephala*+25% corn flour+10% bran pollard; T2=45% *S. bicolor (L.) Moench* + 20% *L. leucocephala* + corn flour 25% + 10% bran pollard; T3=45 % *P. purpureum*+20% *L. leucocephala*+25% corn flour+10% bran pollard. *L. leucocephala*=*Leucaena leucocephala*, *S. bicolor*=*Sorghum bicolor*, *P. purpureum=Pennisetum purpureum*

The research report showed that the water content of meat (%) of male Kacang goats in the T1 treatment was higher (p<0.05) than the T3 treatment. In contrast, the livestock in the T2 treatment had relatively the same water content as T1 and T3 treatments. The collagen content of male Kacang goat in T1 treatment was higher than T2 and T3 (p<0.05); in contrast, the T2 and T3 treatments had relatively the same collagen content. Conversely, the meat cholesterol content of male Kacang goat in T2 and T3 treatments was relatively same and higher (p<0.05) than T1 treatments. The meat acidity (pH) content of male Kacang goat given complete silage made from natural grass, *S. bicolor* (L.) Moench, and *P. purpureum* was relatively similar between treatments. Conversely, the cooking loss (%) tenderness (kg/cm^2^), and water holding capacity (WHC) (%) of male Kacang goats feedlot at T1, T2, and T3 were relatively similar (Table-5).

## Discussion

### Carcass characteristics

#### Slaughter weight

The relatively similar slaughter weight between treatments showed that the use of complete silage made from natural grass, *S. bicolor* (L.) Moench, and *P. purpureum* did not have a positive effect on the slaughter weight of the male Kacang goat being fattened (Table-4). The application of complete silage made from different forage in fattening male Kacang goats could increase body tissue synthesis, thereby contributing to same relative slaughter weight produced in all three treatment groups of animals. Adequate consumption of protein and energy of the three groups of male Kacang goat during the raising phase contributed to the increase in slaughter weight.

If the level of feed consumption in livestock was better during rearing, it could directly influence the growth. Consequently, in a relatively short period of time meat, the growth was optimal and resulted in higher slaughter weight [4]. The slaughter weights obtained in this study were lower than the report of Sumardianto *et al*. [15] which produced 15kg slaughter weights from Kacang goats treated with Angsana leaves (*Pterocarpus indicus*) and Ketapang leaves (*Terminalia catappa*) at a ratio of 50:50%; and Adiwinarti *et al*. [16] conducted a research on male Kacang goats that received CP 14-15% and TDN 56-60%. Differences in slaughter weight in livestock could be influenced by the final weight of raising, the duration of fasting, feed quality, and type of livestock.

#### Carcass and percentage carcass

Carcass weight was relatively the same in T1, T2, and T3 treatments, indicating that the use of complete silage made from different forage did not have a positive effect on the male Kacang goat being fattened. Nevertheless, the carcass percentage of male Kacang goats in T2 and T3 treatments was higher than T1 treatments. The higher weight and percentage carcasses of T2 and T3 compared to T1 treatments caused by enough nutrients obtained by the livestock. Adequate consumption of protein and energy at T2 had a positive impact on the production of carcasses produced. The male Kacang goat in the T1 and T3 treatments had relatively lower growth rates resulting in a carcass and a relatively low percentage of carcasses.

The weight and percentage of a carcass of T2 treatment in this study were relatively the same as the report of Sumardianto *et al*. [15], which obtained a carcass weight of 5.63kg, and the percentage of carcasses was 37.50%. However, the percentage of carcasses in this study was lower than the report of Adiwinarti *et al*. [16], in male Kacang goats that received CP and TDN, respectively, 14-15%, and 56-60%; and the report of Mirdhayati *et al*. [17] which obtained carcass percentage of 43.83±4.9 and 38.88±4.12 in Kacang goats aged 1.5years and more than 1.5years. In addition, the weight and percentage carcass of male Kacang goat in T1 and T3 treatments were lower than the two research reports.

#### Non-carcass

Although the non-carcass weight (kg) was relatively the same between treatments, the non-carcass percentage of T1 treatment was higher than the T2 and T3 treatments (Table-4). This illustrates that the use of natural grass as a basic feed in making complete silage turned out to increase the non-carcass component in male Kacang goats being fattened. The high of non-carcass component had an impact on the lower carcass component produced. According to the observations during slaughtering, the non-carcass component which contributed dominantly was the digestive tract of livestock.

Other than that, the complete silage made from natural grass had slower digestion; thus, it contributed to the slower rate of gastric emptying. Male Kacang goats were fasted for up to 12h before being slaughtered had not contributed much in reducing the contents of the digestive tract, resulting in a higher percentage of non-carcasses produced.

Non-carcass component produced in this study was higher than the report of Adiwinarti *et al*. [18] which produced non-carcasses ranging from 53.20 to 59.21% in Kacang goats fed a protein source that was not degraded in the rumen. The different results of the study illustrated that the variation of non-carcass components of goats could be influenced by the type of feed, the duration of fasting, and age of livestock.

### Meat characteristics

#### Meat chemical quality

Protein content

Research results showed that the meat protein content (%) of male goat feedlot in T1, T2, and T3 treatment was relatively the same and was still in the normal range. This condition illustrated that the use of complete silage made from natural grass, *S. bicolor* (L.) Moench, and *P. purpureum* contributed positively to the meat protein deposit of male Kacang goat. According to Soeparno [19], improving the quality of nutrition can increase the energy used for fat and protein deposition. The intake of CP of these three treatment goats had exceeded the recommendation of Kearl [7] which stated that the CP need for a 15kg BW goat was 48g/head/day. According to Soeparno [19], improving the quality of nutrition could increase the energy used for fat and protein deposition.

According to Lawrie [20], as animals are growing, the rate of protein synthesis and degradation increases, and the rate of protein synthesis often exceeds protein degradation. Conversely, in livestock, which are nearing maturity, the rate of synthesis and degradation protein decreased [21]. Forage feed is generally high in fiber and low in energy, causing a low of the fat carcass content, but it increases protein and water content in meat [22]. The meat protein of this study was relatively the same as the report of Musnandar *et al*. [23] and Ngadiyono *et al*. [4], but lower than the report of Adiwinarti *et al*. [16,18]. The differences between the reports above illustrate that the different types of feed, age, growth phase, and breed of goat influence the meat protein content produced.

Fat content

The results showed that there were variations in meat fat content between treatments, where T3 treatment had a tendency for meat fat content to be higher than other treatments (Table-5). This difference gave an indication that complete silage made from *P. purpureum* had the potential to increase the fat deposits in male Kacang goats being fattened than complete silage made from natural grass. The difference in the fat content of meat could be caused by factors as the type of feed, age, and weight of slaughter. Livestock that consumed high-energy feed contained more fat than livestock that consumed low-energy feed [19]. The fat content of meat in the study was higher than the report of Adiwinarti *et al*. [16,18] and the report of Musnandar *et al*. [23]. This difference illustrated that the giving of complete silage to goats contributed to the increase in meat fat content. However, the meat fat in this study was relatively the same as the report of Ngadiyono *et al*. [24].

Variations of fat content in meat could be influenced by many factors. Differences in livestock genotyping had an impact on differences in meat quality [25], as well as breed, age, species, muscle location, and feed [26]. Increased concentrations of feed energy and/or protein/energy ratio in feed and the use of volatile fatty acids, especially propionate for gluconeogenesis, could have an impact on changes in the meat chemical components, especially fat content of carcass [21].

Water content

The meat water content (%) of male Kacang goat at T1 treatment in this study was higher than T3 treatment (Table-5). The higher water content of meat in the T1 treatment had a close relationship with the relatively low fat of meat content of T1 treatments compared to the fat content in T3. In contrast, T1 and T2 had a lower and relatively similar water content of meat because the fat content was not too different either.

Although there were variations in the water content of meat between T1, T2, and T3 treatments, in general, the water content obtained in this study was still within the normal range. Theoretically, meat water content has a negative relationship with the fat content of meat where the difference in water content of the meat is influenced by intramuscular fat (IMF) content. If the meat content is high, the meat fat content is low, and vice versa. Muscle water content frequently has a significant negative relationship with meat fat content [21].

The water content in this study was lower than the report of Musnandar *et al*. [23] and Ngadiyono *et al*. [24], but it was relatively the same as the report of Adiwinarti *et al*. [16,18]. The water component in the tendon of mammalian after rigor mortis could reach 75% [20]. This difference was related to fat deposits in livestock fattened. Nutritional deficiencies in livestock could increase the level of the water content of the meat and the percentage of intramuscular collagen so that the meat got harder [21].

Collagen content

The use of natural grass as a basic feed in making complete silage has an effect in increasing meat collagen (Table-5) compared to the use of *S. bicolor* (L.) Moench and *P. purpureum*. The results of this study indicated that the use of *S. bicolor* (L.) Moench and *P. purpureum* as the basis for making complete silage contributed positively to reduce meat collagen. Conversely, complete silage made from natural grass produced higher collagen meat.

The collagen levels of meat can differ between sex and ages and between meats in the same carcass. Meat collagen levels are influenced by fat content. Relatively high-fat content will dissolve or reduce collagen content [19]. The results of this study were in line with the above opinion because the goats in T2 and T3 treatments had higher fat content than T1, which had lower collagen content. In contrast, the male Kacang goats in T1 treatments which had a low meat fat content had higher collagen.

The collagen content of male goats which obtained complete silage made from *S. bicolor* (L.) Moench and *P. purpureum* in this study was lower than the report of Adiwinarti *et al*. [16,18]. Conversely, male goats that obtained complete silage made from natural grasses had higher collagen content. It showed that the application of complete silage made from *S. bicolor* (L) Moench and *P. purpureum* contributed to low levels of meat collagen. Nevertheless, this report was higher than the report of Hwang *et al*. [27] of 0.67±0.18 and 0.90±0.16, who conducted research on Korean local black goat.

Cholesterol content

Meat cholesterol in goat is one of the causes of health problems of humans. Arun *et al*. [28] reported that the meat cholesterol content of goat, which reached 3.2mg/g could contribute to increased health problems. The results of the study showed that the meat cholesterol content of male Kacang goat in T2 and T3 treatments had cholesterol content, which was relatively the same and higher (p<0.05) than T1 treatments.

It indicated that the use of complete silage made from *S. bicolor* (L.) Moench and *P. purpureum* contributed to increase meat cholesterol. This high cholesterol content had a positive correlation with meat fat produced by T2 and T3, which was higher than T1.

Cholesterol, in this study, was higher than the report of Suryanto *et al*. [29] that obtained cholesterol ranging from 30.13 to 34.96mg/100g in Kacang goats given fermented cocoa skin, but lower than the report of Mirdhayati *et al*. [17] that obtained cholesterol in Kacang goat meat ranged from 112 to 256mg/100g. Cholesterol levels can differ between livestock, and this difference can be caused by differences in food type, age of livestock, and management [29].

### Physical quality of meat

#### Acidity (pH)

The meat acidity (pH) content of male Kacang goat given complete silage made from natural grass, *S. bicolor* (L.) Moench, and *P. purpureum* was relatively similar between treatments, which was still within the normal range (Table-5). Thus, all three treatments had the same contribution to the pH content of the meat.

A reservoir of muscle glycogen is one of the factors that also influence the level of ultimate pH. In this study, the muscle glycogen reserves owned by the goats in the aforementioned three treatments that were considered to be not much different so that it affected the same relative to the pH of the meat. Meat has a pH ultimate in the range of 5.5-5.8, in which the pH amount of the meat or carcass ultimate depends on the amount of muscle energy reserves when the goats were slaughtered. The more glycogen reserves at the time of the slaughter, the highest pH achieved will be lower [19].

The acidity level of Kacang goat meat in this study was lower than the report of Adiwinarti *et al*. [16,18], which obtained the pH of male Kacang goat ranging from 6.05 to 6.08 and 6.01. The final pH value of the meat achieved can provide clues for good meat quality. Meat that has a normal pH between 5.5 and 5.7 gives a bright red color [20].

According to Soeparno [19], the rise and fall of pH postmortem of meat are influenced by intrinsic and extrinsic factors. Intrinsic factors that influence include species, muscle type, muscle glycogen, and variability among livestock. Conversely, extrinsic factors include environmental temperature, treatment of additives before slaughter, and stress before slaughter.

#### Cooking loss

In general, cooking losses in the research were within the normal range and were quite low. The low cooking losses in T1, T2, and T3 treatments were related to the water capacity of the meat, which was apparently still quite high with a range of 33.91-34.67%; and the ultimate pH value of the meat was 5.28-5.45.

The normal range cooking loss of meat from T1, T2, and T3 treatments illustrated that the nutrients in meat were still well protected (protected) during the cooking process. Adequate consumption of feed energy from T2 and T3 had an impact on the formation of IMF (marbling), which was quite high. As a result of the cooking process, there was quite a lot of liquid protected, so the quality of meat was maintained properly. According to Soeparno [19], high IMF formation will inhibit the discharge of fluids during the cooking process. The value of cooking losses was closely related to the type of feed consumed by the livestock.

The livestock that consume forages with low digestibility have relatively little formation of fat, especially marbling so that cooking losses increase [19]. The amount of cooking losses is influenced by the amount of cellular membrane damage, the amount of water comes out of the meat, the shelf life of the meat, protein degradation, and the ability of the meat to bind water [30]. The cooking loss of this research result was lower than the report of Adiwinarti *et al*. [18], but it was relatively the same as the report of Adiwinarti *et al*. [16].

#### Tenderness

The same relatively and moderate meat tender of T1, T2, and T3 treatments were related to the higher water holding capacity and lower cooking losses of three treatments. In addition, the tenderness of meat at T1, T2, and T3 treatments was also related to the high IMF content of third treatments, besides the lower collagen content, specifically the T2 and T3 treatments.

Meat tenderness is determined by three meat components, namely, myofibril structure and contraction status, connective tissue content and the degree of cross-linking, and water holding capacity by protein, and meat juices [19]. According to Pearson and Dutson [31], the range of shear force measurements of meat tenderness is divided into three: Soft, quite soft, and tough with the values range of 0-3, 3-6, and 6-11kg/cm^2^. If the shear force measurement results show more than 11, then it is difficult for humans to eat the meat.

Goat meat tenderness of this study was lower than reports made by Hwang *et al*. [27] who obtained meat tenderness ranging from 3.92±0.14 to 4.05±0.16 (kg/cm^2^); Adiwinarti *et al*. [16,18] and Lopes *et al*. [26]. Fattening beef cattle using forages or concentrates produce carcasses with tenderness values that do not differ from each other [32]. According to Soeparno [19], the high breaking strength value illustrates that meat produced by livestock is getting tougher. On the other hand, the diminishing value of breaking meat illustrates that meat produced by livestock is getting tender.

According to various reports, the main factors influencing the level of meat tenderness are the amount of collagen and the level of collagen solubility [20]. Thus, meat collagen contributes greatly in determining the softness of meat from livestock. Related to the above opinion, the meat in the T2 and T3 treatments had lower collagen content, thus contributing to the more tender meat produced. According to Soeparno [19], different collagen denaturation also determines the difference in the value of meat breaking. In addition, differences in the nature of collagen fibers were also affected by their solubility.

#### Water holding capacity (WHC)

Water holding capacity is a significant parameter examined and controlled by meat industry. Changes in water holding capacity are very sensitive indicator for changes in the charges and structure of myofibrillar protein [33]. The results of this study showed that, in general, the provision of complete silage made from natural grass, *S. bicolor* (L.) Moench, and *P. purpureum* on male Kacang goats had a positive impact on the water holding capacity of the meat. It means that the use of different forages as a basic material in making complete silage does not affect the water holding capacity.

Water holding capacity is the power of meat to hold water due to the influence of external factors, especially the loss of water, fat, and ions during cooking [25]. The water holding capacity can be determined by several factors, including glycolysis postmortem [25], pH [19,20,25], and carcass withering [25].

Theoretically, the ultimate pH of meat has an important role in determining WHC. The greater decrease in pH of carcass (postmortem) will affect the water holding capacity. If the final pH of the meat is higher, it then reduces the water holding capacity of the meat [20,22]. The increasing or decreasing pH of the meat from the isoelectric point has an impact on increasing the water holding capacity by creating an unbalanced charge [19]. According to Watanabe *et al*. [34], IMF is not significantly correlated with WHC, and the correlation between pH and WHC is higher. Therefore, it is suggested that it is difficult to improve WHC by only increasing IMF content, and pH control is more important for improving WHC than IMF content.

The younger age of the animals was also considered to have an effect on the water holding capacity to the meat, which was not different between the treatments. According to Soeparno [19], the age factor also influences the water holding capacity. The young sheep or lamb, for example, tends to have a higher WHC than the older sheep.

## Conclusion

The use of *S. bicolor* (L.) Moench and *P. purpureum* as a basic forage in making complete silage has been proven to increase the carcass percentage, the content (%) of fat, collagen, and cholesterol. Otherwise, the content of pH, tenderness (kg/cm^2^), cooking loss (%), and water holding capacity (%) of male Kacang goat fattened does not have a positive influence. Therefore, *S. bicolor* (L.) Moench and *P. purpureum* plants have great potential to be developed by farmers/ranchers as the feed for goats.

## Authors’ Contributions

PKT conducted experimental design of the research, data collection, reporting, and drafting of the article. GFB conducted processing of complete silage, supervised the research, and data analysis. The authors read and approved the final manuscript.
